# An Optimized Workflow for the Discovery of New Antimicrobial Compounds Targeting Bacterial RNA Polymerase Complex Formation

**DOI:** 10.3390/antibiotics11101449

**Published:** 2022-10-21

**Authors:** Alessia Caputo, Sara Sartini, Elisabetta Levati, Ilaria Minato, Gian Marco Elisi, Adriana Di Stasi, Catherine Guillou, Peter G. Goekjian, Pierre Garcia, David Gueyrard, Stéphane Bach, Arnaud Comte, Simone Ottonello, Silvia Rivara, Barbara Montanini

**Affiliations:** 1 Laboratory of Biochemistry and Molecular Biology, Department of Chemistry, Life Sciences and Environmental Sustainability, University of Parma, 43124 Parma, Italy; 2 Interdepartmental Research Centre Biopharmanet-Tec, University of Parma, 43124 Parma, Italy; 3 Department of Orthopaedic Surgery, David Geffen School of Medicine, University of California Los Angeles (UCLA), Los Angeles, CA 90095, USA; 4 Department of Food and Drug, University of Parma, 43124 Parma, Italy; 5 Department of Life Sciences, University of Trieste, 34127 Trieste, Italy; 6 Centre de Recherche de Gif, Institut de Chimie des Substances Naturelles, CNRS, 1 Avenue de la Terrasse, 91198 Gif-sur-Yvette, France; 7 Laboratoire Chimie Organique 2 Glycochimie, ICBMS UMR 5246 CNRS-Université Claude Bernard Lyon 1, Université de Lyon, 69622 Villeurbanne, France; 8 Sorbonne Université, CNRS, UMR 8227, Integrative Biology of Marine Models, Team Physiology and Cell Fate, Station Biologique de Roscoff, CS 90074, 29680 Roscoff, France; 9 Sorbonne Université, CNRS, FR 2424, Plateforme de criblage KISSf (Kinase Inhibitor Specialized Screening Facility), Station Biologique de Roscoff, 29680 Roscoff, France; 10 Centre of Excellence for Pharmaceutical Sciences, North-West University, Private Bag X6001, Potchefstroom 2520, South Africa; 11 Chimiothèque, ICBMS UMR 5246 CNRS-Université Claude Bernard Lyon 1, Université de Lyon, 69622 Villeurbanne, France

**Keywords:** yeast Bioluminescence Resonance Energy Transfer (yBRET), protein–protein interaction (PPI), protein–protein interaction inhibitor, bacterial RNA polymerase (RNAP), RNAP holoenzyme assembly, bacterial transcription inhibitors, antibiotics, drug-discovery workflow

## Abstract

Bacterial resistance represents a major health problem worldwide and there is an urgent need to develop first-in-class compounds directed against new therapeutic targets. We previously developed a drug-discovery platform to identify new antimicrobials able to disrupt the protein–protein interaction between the β’ subunit and the σ^70^ initiation factor of bacterial RNA polymerase, which is essential for transcription. As a follow-up to such work, we have improved the discovery strategy to make it less time-consuming and more cost-effective. This involves three sequential assays, easily scalable to a high-throughput format, and a subsequent in-depth characterization only limited to hits that passed the three tests. This optimized workflow, applied to the screening of 5360 small molecules from three synthetic and natural compound libraries, led to the identification of six compounds interfering with the β’–σ^70^ interaction, and thus was capable of inhibiting promoter-specific RNA transcription and bacterial growth. Upon supplementation with a permeability adjuvant, the two most potent transcription-inhibiting compounds displayed a strong antibacterial activity against *Escherichia coli* with minimum inhibitory concentration (MIC) values among the lowest (0.87–1.56 μM) thus far reported for β’–σ PPI inhibitors. The newly identified hit compounds share structural feature similarities with those of a pharmacophore model previously developed from known inhibitors.

## 1. Introduction

The discovery and widespread clinical use of antibiotics have transformed modern medicine. Antibiotics are key drugs not only to treat common infections but also for cutting-edge surgical procedures, organ transplantation, and management of cancer patients, among others [1].

Although antibiotic resistance can occur naturally, it is sped up by antibiotics misuse in humans and livestock and nowadays is among the leading causes of all-age death. As a result of the spreading of antibiotic resistance among common pathogens, ordinary diseases are becoming increasingly unresponsive to first-line antibiotics and lifesaving medical procedures are riskier to perform.

The “antibiotic crisis” is aggravated by the difficulty of identifying and developing new classes of antibacterial drugs. Most antibiotics that have reached the market in recent years are chemical derivatives of preexisting antibacterial compounds, whose targets have been shown to be susceptible to resistance mechanisms [2].

In recent years, protein–protein interactions (PPIs) have been recognized as attractive targets for antimicrobial drug development since compensatory mutations on both interaction partners are required to confer resistance while preserving bacterial cell viability [3]. 

**Figure 1:**
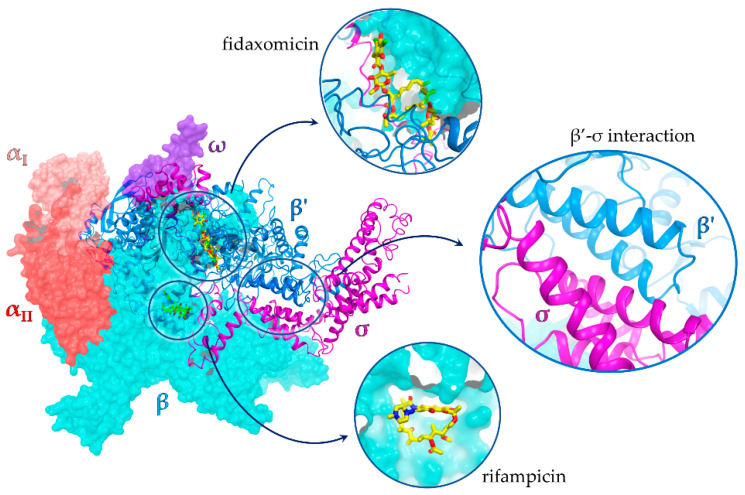
X-ray structure of the *E. coli* RNA polymerase σ^70^ holoenzyme complex (PDB id 4YG2 [11]) with an expanded view of the β’ subunit–σ^70^ PPI; binding sites of rifampicin and fidaxomicin are also shown. Rifampicin and fidaxomicin structures are from PDB structures 1YNN [20] and 7L7B [21], respectively.

The main processes targeted by current antibiotics are DNA replication, mRNA translation, and cell wall biosynthesis [4]. To date, the only two classes of antibiotics approved for clinical use acting on bacterial transcription are rifamycins and lipiarmycins (fidaxomicin). Rifamycins bind to a region close to the RNA polymerase (RNAP) active site, while lipiarmycins allosterically inhibit RNAP binding to template DNA (Figure 1) [5,6,7]. However, both are susceptible to resistance [8,9]. 

Transcriptional regulation requires the interaction of RNAP with multiple accessories, yet functionally crucial factors, among which are the σ factors, are essential for transcription initiation and bacterial cell viability. The RNAP core enzyme (RNAPc) is composed of five subunits (α_I_, α_II,_ β, β’, and ω), and upon binding of the σ factor, it is converted to the holoenzyme form (RNAPh) that specifically recognizes promoter elements and induces DNA strand separation with exposure of the transcription starting site. The RNAPc–σ interface involves multiple RNAP domains, but the key interaction is the one taking place between the β’ subunit clamp helix (CH) domain and the 2.2 region of σ (σ2.2). Housekeeping σ factors (e.g., σ^70^ in gram-negative bacteria such as *Escherichia coli* and σ^A^ in gram-positive bacteria such as *Bacillus subtilis*) are highly conserved among bacteria and have no counterpart in eukaryotic organisms. This makes the PPI interface between the RNAP β’ CH domain and σ factor a promising target for the development of broad-spectrum antibiotics, which are expected to selectively interfere with bacterial DNA transcription with no (or very little) adverse effects on eukaryotic host cells [10]. The availability of high-resolution structures of RNAPh complexes [11,12,13,14,15] has promoted the structure-guided discovery of antimicrobial agents targeting the RNAP–σ factor interaction [16,17,18,19].

Initial attempts to identify β’–σ interaction inhibitors led to the identification of two compounds, GKL003 (**1**, Supplementary Figure S1) and C5 (**2**, Supplementary Figure S1), that bind the β’ subunit and interfere with in vitro transcription initiation at nanomolar concentrations [6,16]. However, one of the biggest challenges faced by all antibiotic candidates is bacterial cell penetration and target accessibility. This is especially relevant for outer membrane-containing gram-negative bacteria [5]. Indeed, limited internalization and/or solubility issues are probably the main reasons why compounds **1** and **2** required concentrations 500−100,000-fold higher than those effective in vitro when tested for their ability to inhibit bacterial growth [16,17]. To overcome these problems, smaller compounds based on the indole structure of inhibitor **1** were designed, yielding 1*H*-indole-7-glyoxylamides with significant antibacterial potency at much lower concentrations (e.g., compound **3**, Supplementary Figure S1, with an MIC of 3.1 μM against *Staphylococcus aureus*) [19,22]. 

In vitro high-throughput assays, including a Luminescence Resonance Energy Transfer-based assay (LRET) and an ELISA dissociation assay, were employed to discover new inhibitors of the β’–σ^70^ interaction, but hit compounds thus identified displayed only limited or no antimicrobial activity, possibly due to a low internalization capacity [23,24,25].

We recently developed a Bioluminescence Resonance Energy Transfer assay in yeast (yBRET) as an in vivo tool for the screening of large candidate compound libraries [26] aimed at the discovery of novel RNAP β’–σ^70^ PPI inhibitors [18]. Due to its intracellular mode of operation, yBRET, utilized as a first screening step, facilitates the identification of potentially cell-permeant compounds devoid of toxicity against eukaryotic cells. In a first screening, seven hits were retrieved from a library of 5000 compounds, four of which proved capable of binding the RNAP β’ subunit and inhibiting the interaction of interest. Compounds belonging to the class of indol-3-yl-ureas were found to be as one of the most potent. In particular, compound **4** (Supplementary Materials, Figure S1) was shown to inhibit RNAP transcription in vitro (IC_50_ = 28.6 μM) and displayed antibacterial activity against the gram-positive bacteria *B. subtilis*, *S. aureus*, and *Listeria monocytogenes* as well as against *E. coli* upon supplementation with a membrane permeabilizer, with MIC values in the 25–100 μM range [18]. 

Here, we optimized our yBRET-based discovery platform, especially with regard to its overall productivity and hit identification/validation efficiency. To this end, the updated workflow was applied to the search for novel inhibitors of the β’–σ^70^ interaction through the screening of three small-molecule libraries comprising a total of 5360 compounds. Six hit compounds, belonging to three different chemical classes, were identified, and were further investigated for their ability to inhibit RNAP-dependent in vitro transcription and bacterial growth. One compound completely suppressed in vitro transcription (IC_50_ = 14 μM), while two of them, supplemented with the membrane permeabilizer polymyxin B nonapeptide (PMBN), inhibited *E. coli* growth at (sub)micromolar concentrations (MIC 0.87–1.56 μM). The structures of the most potent, newly identified inhibitors were analyzed in light of our previously developed pharmacophore model [18].

## 2. Results and Discussion

### 2.1. Drug-Discovery Workflow

**Figure 2:**
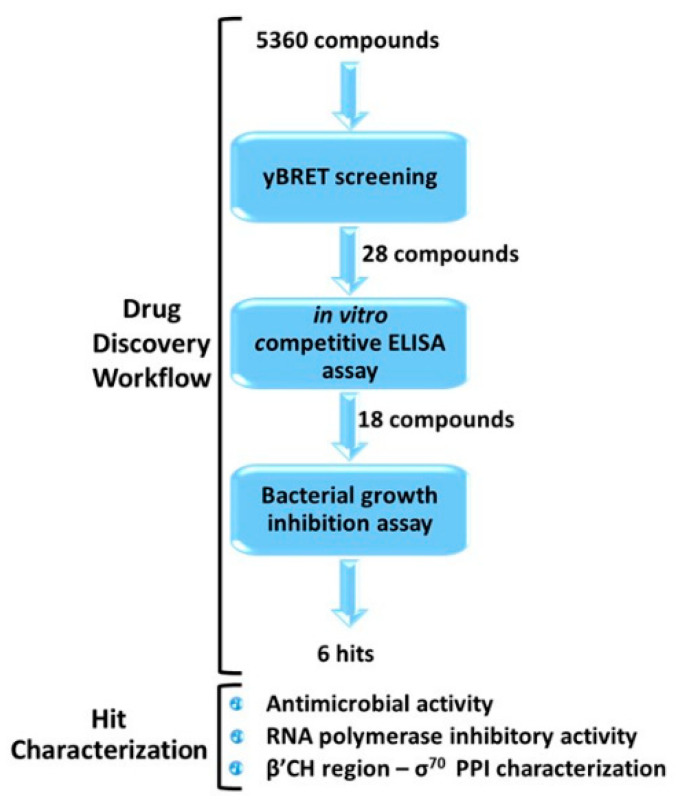
Outline of the drug-discovery workflow applied to the identification of small-molecule inhibitors of RNAP β’–σ^70^ interaction, endowed with antimicrobial activity. The number of compounds that passed each step is indicated. Candidate hits were further characterized with the indicated assays, as described in the following sections.

Our optimized workflow relies on three sequential assays, which are easily scalable to a high-throughput format: (1) yBRET assay for a first in vivo screening of large compound libraries in yeast cells in order to identify candidate hits while counter-selecting molecules toxic to eukaryotic cells; (2) competitive ELISA to validate candidate hit ability to inhibit the β’–σ^70^ PPI in vitro; (3) bacterial growth inhibition assay to test the antimicrobial potential of the selected molecules. All assays were conducted at a fixed compound concentration and only hits that passed the three tests were subjected to a more detailed characterization (Figure 2 and Figure S2).

In this work, the optimized drug-discovery workflow was applied to the screening of 5360 molecules derived from three libraries:

(i) ReCC, Roscoff essential Chemical Collection (Roscoff, France) [27,28], an in-house library of putative kinase inhibitors (1040 small synthetic compounds).
(ii) The ICBMS-Lyon 1 University in-house library, containing 3120 original synthetic and natural compounds, selected for their scaffold diversity and physicochemical properties.
(iii) The PKRC library, containing 1200 synthetic and natural compounds, focused on protein kinase inhibitors [29].

#### 2.1.1. yBRET Screening

The first step of our drug-discovery workflow is a BRET-based assay conducted in yeast using 96-well plates, with a typical (but easily scalable) screening throughput of up to 800 compounds/day. In addition to a very efficient first selection of candidate PPI inhibitors, yBRET offers two further advantages: (i) it can be designed to identify inhibitors that preferentially target one of the two interacting partners, and (ii) it allows the early counter-selection of compounds with cytotoxic effects on eukaryotic cells. Moreover, since the interacting protein partners are expressed inside yeast cells, candidate compounds must cross both the yeast cell wall and plasma membrane in order to reach their target. In fact, one of the main drawbacks of the first reported β’–σ interaction inhibitors, identified through an in vitro screening, was their low antimicrobial activity, despite in vitro inhibitory concentrations in the nanomolar range [16,17]. Although the yeast cell envelope differs from that of bacterial cells, it nonetheless represents a physical barrier to compound internalization, and yBRET-positive hits have, thus, a greater chance of being able to penetrate bacterial cells compared to compounds primarily selected by in vitro assays. 

As previously described [19], for yBRET screening platform set-up, we reproduced the interaction between the CH region of β′ (aa 1–334) and full-length σ^70^, arranged as an acceptor- and a donor-fusion protein pair capable of generating a BRET signal upon interaction reconstitution. The β′ target fused to the Yellow Fluorescent Protein (YFP) was used as a constitutively expressed acceptor-fusion partner to allow the inhibitor to bind it before complex formation and PPI establishment, facilitating the identification of compounds with limited binding affinity, typically present in screening libraries. The donor-fusion partner, in our case σ-NLuc, was expressed under the control of the galactose-inducible GAL1 promoter in the hyperpermeable Δ*erg6* yeast strain to maximize the uptake of the luciferase substrate and small-molecule compounds as well [18].

The 5360 compounds were screened at a fixed concentration of 20 µM. Forty-five molecules reduced the yBRET signal by at least 15% and were re-assayed in a secondary screening at both the same and at a lower concentration (10 µM). A compound-induced drop of the NanoLuc luciferase signal to less than 50% of the control (i.e., the signal measured in the presence of the DMSO solvent but without any added compound) was considered indicative of yeast “sickness”. Based on this criterion, seventeen compounds were labeled as potentially toxic to eukaryotic cells and discarded. This led to the identification and retention of 28 hit molecules: four belonging to the ReCC library, 14 to the ICBMS library, and five to the PKRC library.

#### 2.1.2. In Vitro Competitive ELISA Assay

An orthogonal in vitro assay based on a different readout (i.e., competitive ELISA) was used as an independent validation tool to validate the ability of yBRET-identified compounds to actually inhibit the β’–σ^70^ PPI. In this in vitro set-up, individual candidate molecules (each at 100 µM concentration) were incubated with β’ before the addition of σ^70^ and assessment of interaction reconstitution with an antibody directed against the σ^70^ fusion tag. Eighteen of the 28 yBRET-positive compounds were thus confirmed as true inhibitors of the β’–σ^70^ PPI (i.e., they inhibited the interaction by at least 50%); their structures and percentage inhibition values are reported in Table S1.

There are many possible reasons for discrepancies between yBRET and ELISA assay results; for example, false-positive compounds may directly interfere with the BRET signal (e.g., by adsorbing the light emitted by the donor protein), or interact with unrelated cellular components.

#### 2.1.3. Bacterial Growth Inhibition Assay

To avoid the characterization of hit compounds lacking antibacterial activity, we subsequently tested the ability of the 18 ELISA-validated compounds to inhibit bacterial growth, using the previously identified indol-3-yl-urea derivative **4** as a reference [18]. Two nonpathogenic bacteria were employed for this assay: the gram-positive *B. subtilis* (WB800N strain) and the gram-negative *E. coli* (DH10T1R strain). Bacterial growth was monitored in the presence of a fixed concentration of each compound (100 µM) and six molecules were found to inhibit growth of at least one of the two tested strains by more than 20%. The remaining twelve compounds might have been unable to cross the bacterial membrane or were modified and/or extruded from the cell. Consistent with the eukaryotic cytotoxicity counter-selection criterion applied in the yBRET screening, none of these six hit compounds displayed any appreciable growth inhibition in the unicellular eukaryote *Saccharomyces cerevisiae* (Table 1). By comparison, when assayed under the same conditions, 10 of the 17 compounds that were found to interfere with yeast viability in the primary yBRET screening (see Section 2.1.1.) inhibited yeast growth by 28–37%. This additional assay, which was conducted on a wild-type (W303) rather than on the hyperpermeable Δ*erg6* strain, is not part of our streamlined antibiotic discovery workflow and was only aimed at verifying the cytotoxicity prediction reliability of yBRET.

Compounds **5** and **7**, belonging to the same pyrido-pyrrolo-isoquinoline chemical class, displayed a preferential inhibitory activity against *E. coli*, whereas the other compounds were similarly effective against gram-positive and gram-negative bacteria. No compound inhibited *E. coli* growth without addition of the outer membrane permeabilizer PMBN, suggesting a common deficiency in outer membrane penetration and internalization capacity. As shown here and as pointed out by many previous studies in which hyperpermeable *E. coli* mutants were used for antibacterial activity assays (e.g., [30,31]), inhibitor internalization likely represents the main bottleneck in the discovery of new effective antibiotics. 

The six positive hits were then further characterized for antimicrobial activity, ability to inhibit in vitro transcription, and binding features. 

### 2.2. Hit Characterization

A series of orthogonal assays included in the “hit characterization” phase (Figure 2) was also part of our previously reported antibiotic discovery workflow [18]. The major difference regards the choice and order of utilization of the initial selection assays. In particular, all the functional characterization steps, including MIC determination, bacterial growth curves, transcription inhibition, and ELISA dose–response assays, are only performed after a preliminary evaluation of the bacterial growth inhibition potential of individual candidate hit compounds. In fact, while the assays utilized for the three main steps of our streamlined workflow are easily adaptable to a high-throughput screening format, the subsequent characterization assays (including additional ELISAs to determine target selectivity and compound binding affinity) are much more time-consuming and costly. The advantage of this modified experimental set-up obviously increases with the size of the starting library and the number of candidate hit compounds identified by the yBRET screening.

#### 2.2.1. Antimicrobial Activity

**Figure 3:**
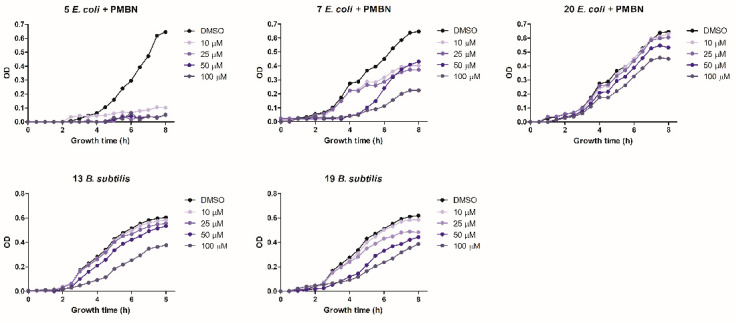
Antibacterial activity determined at increasing growth times and at different concentrations of compounds **5**, **7**, and **20** (*E. coli*) and compounds **13** and **19** (*B. subtilis*) as indicated. Data are the mean of three replicates.

The antibacterial activity of the six positive hits was subsequently assayed by monitoring growth inhibition at different compound concentrations. The goal of this assay was to assess the persistence of antimicrobial activity over time at different, lower concentrations. Compound **5** completely inhibited *E. coli* growth for the entire observation time (8 h) even at the lowest (10 µM) concentration. Compound **7** was less potent but maintained a significant (38%) growth inhibition at the lowest concentration (10 µM), whereas compound **20** was only partially effective at a 100 µM concentration. Compounds **13** and **19** significantly inhibited *B. subtilis* growth but only at a 100 µM concentration. Compounds **7** and **19** almost completely inhibited *E. coli* and *B. subtilis* growth, respectively, at a 50 µM concentration, but their antibacterial activity only lasted for 4 h (Figure 3). Compound **9** failed to exert any appreciable inhibitory activity on either bacterium at concentrations ≤ 100 µM, while compound **13**, tested at concentrations ≤ 100 µM, did not inhibit the growth of *E. coli* (not shown).

We also determined the MIC values against *B. subtilis* WB800N and *E. coli* DH10T1R. Consistent with the results of growth inhibition assays, compounds **5** and **7** were the most potent against *E. coli*, with MIC values of 1.56 µM and 0.78 µM, respectively, when supplemented with the PMBN permeabilizer, but did not inhibit *B. subtilis* growth (Table 1). Both compounds were more active than reference compound **4** against *E. coli*, with MIC values among the lowest thus far reported in the literature. Compound **20** displayed a MIC value of 12.5 µM against *E. coli*, but much higher MIC values against *B. subtilis* (Table 1).

#### 2.2.2. RNA Polymerase Inhibitory Activity

Compounds endowed with antibacterial activity were further characterized for their ability to inhibit promoter-specific RNA transcription in vitro. 

**Figure 4:**
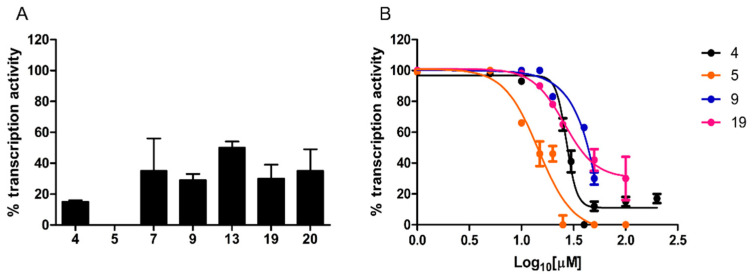
Transcription inhibition capacity. (**A**) In vitro transcription activity was measured under single-round conditions in the presence of a fixed concentration (100 µM) of the indicated hit compounds and of reference compound **4**. (**B**) Dose–response curves determined for a selected subset of compounds. In vitro transcription activity is reported as percent relative to the DMSO vehicle. Data are the mean of four replicates; error bars represent SD.

A single-round transcription assay was first performed at a fixed, 100 µM concentration. As shown in Figure 4A, compound **5** completely inhibited transcription, whereas a residual transcriptional activity, ranging from 29% to 50% of the untreated control, was observed with the other compounds. 

Dose–response curves were determined for compounds inhibiting transcription by at least 70%. As shown in Figure 4B, while compound **9** yielded an IC_50_ of 71 ± 2.46 µM, significantly lower values (Mann–Whitney test, *p* < 0.0001), comparable to the IC_50_ of reference compound **4** (27 ± 1.8 µM), were determined for compound **5** (IC_50_ = 14.5 ± 1.9 µM) and **19** (IC_50_ = 25.75 ± 1.85 µM). 

An important difference with our previous screening platform [18], in which a radiolabeled nucleoside triphosphate was employed as tracer for in vitro transcription, is the use of a more convenient/safer fluorescence-based assay that does not require reaction products fractionation on polyacrylamide gels. The reliability of this assay was confirmed by the nearly identical IC_50_ values determined for reference compound **4** with the fluorescence-based and the previous radioactive assay (27 µM and 28 µM, respectively).

#### 2.2.3. β’ CH Region–σ^70^ PPI Inhibition Characterization

Although the yBRET assay design was meant to favor the selection of β’ targeting compounds, it was still possible that some of the positive hits capable of inhibiting transcription might do so by binding to σ^70^. 

We tested this possibility with a “reverse” competitive ELISA, in which transcription-inhibiting hit compounds were incubated with σ^70^ (instead of β’) as the primary interactor; compound **4**, previously shown to inhibit the β’–σ^70^ interaction by specifically targeting the β’ subunit [18], served as a reference also for this assay.

None of the tested compounds inhibited the β’–σ^70^ interaction under these “reverse” assay conditions, thus confirming that they all bind specifically to the β’ subunit, in agreement with the yBRET assay design, in which β’ was constitutively expressed at the beginning of the assay, while σ^70^ expression was induced later on, after compound addition.

The binding inhibition capacity of the six hits was then measured in a dose–response ELISA. Compounds **5**, **7**, **9**, and **13**, with IC_50_ values of 15.11 µM, 8.56 µM, 13.93 µM, and 16.35 µM, respectively, displayed a potency similar to that of reference compound **4** (IC_50_ of 8.09 µM), whereas compounds **19** and **20**, which yielded IC_50_ values of 40 µM and 80 µM, were 5- to 10-fold less potent, respectively (Table 1).

### 2.3. Similarity with Indolyl-Urea Inhibitors and Pharmacophore Model Matching

**Figure 5:**
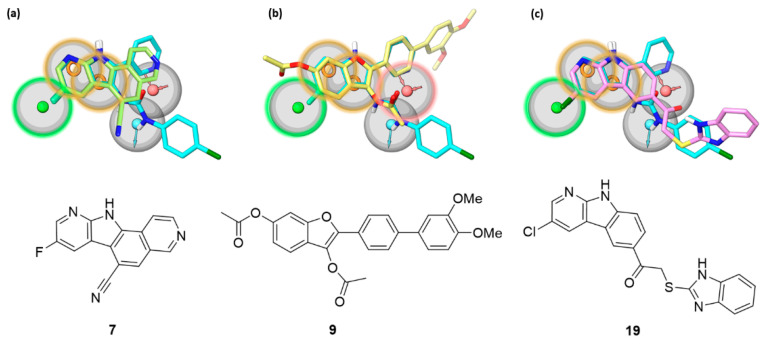
The most potent β’–σ interaction inhibitors belonging to (**a**) the pyrido-pyrrolo-isoquinoline (**7**), (**b**) the benzofuran (**9**), and (**c**) the pyrido-indole (**19**) series are shown, superposed to the reference indolyl-urea inhibitor **4** (cyan carbons) fitted on the pharmacophore model previously devised for β’–σ interaction inhibitors [18]. The pharmacophore model comprises hydrophobic (green), aromatic (orange), and hydrogen bond donor (light blue) and acceptor (red) features characterized by a tolerance of 2 Å (gray sphere). The features highlighted with a colored contour of the tolerance region represent the sites matched by each of the new inhibitors.

The newly identified inhibitors belong to three different chemical classes (i.e., pyrido-pyrrolo-isoquinolines **5** and **7**, benzofurans **9** and **13**, and pyrido-indoles **19** and **20**), that share structural elements with the indol-3-yl-urea derivatives, previously retrieved by a yBRET screening as β’–σ^70^ interaction inhibitors targeting the β’ subunit [18]. Indolyl-ureas were identified as the most potent class of PPI inhibitors among a set of structurally different compounds of the “Open Collection Scaffolds” library from Compounds Australia (Griffith University) [32]. The most potent inhibitors, together with literature reference compounds such as **1** [16], were used to generate a pharmacophore model recapitulating the chemical properties and spatial requirements for interaction with the β’ subunit. The 5-points pharmacophore model is characterized by two aromatic features and a hydrophobic one, and by an acceptor and a donor feature roughly perpendicular to the plane occupied by the former three hydrophobic elements (Figure 5) [18]. The pharmacophore model allowed a binding site on the tip of the β’ CH region to be hypothesized in which the planar aromatic portion of the compounds lies on the hydrophobic surface near to the helix-turn-helix motif of β’, while the polar substituents undertake hydrogen bonds with neighboring residues.

The newly identified pyrido-pyrrolo-isoquinoline, benzofuran, and pyrido-indole inhibitors share with indolyl-ureas the presence of a planar aromatic portion. In pyrido-pyrrolo-isoquinolines the central benzene ring of the tetracyclic structure fuses the indole portion to the pyridine ring, the same structural elements present in indolyl-ureas (e.g., compound **4**). Benzofurans carry an aromatic substituent in position 2, recalling the 2-pyridine ring of indolyl-ureas. β’–σ PPI inhibitors characterized by a benzofuran scaffold have already been reported as bioisosteres of indole derivatives obtained from a structural simplification of compound **1** [22]. Pyrido-indoles carry an extended tricyclic scaffold, compared to indolyl-ureas, and are mostly functionalized with bulky substituents in position 5 of the indole ring. The structural similarity of the new inhibitors with indolyl-ureas can be appreciated in Figure 5, in which the most potent β’–σ inhibitor (ELISA assay) from each series was submitted to a shape screening similarity to the reference indolyl-urea **4** arranged according to the pharmacophore model. The three compounds share with indolyl-ureas the aromatic portion, further enlarged in polycyclic rings. Additionally, the carbonyl oxygen atom belonging to the 3-acetoxy substituent of benzofurans is able to fit the hydrogen bond acceptor feature, in the same way as the carbonyl group of the urea portion of reference compound **4**.

## 3. Conclusions

This work presents an optimized workflow for the discovery of new antibiotics acting as inhibitors of the RNAP β’–σ PPI. The workflow relies on the sequential utilization of three assays, easily scalable to a high-throughput format, and was subjected to a proof-of-concept validation based on the screening of 5360 small molecules, which led to the identification of six new antibacterial compounds, which widen the number of chemical structures able to interfere with β’–σ interaction. 

Although satisfactory as the result of a preliminary screening campaign, the activity of the six hit compounds needs to be improved through medicinal chemistry optimization programs that should primarily be tailored to (i) increase target binding affinity, (ii) favor bacterial cell wall/membrane penetration, and (iii) remove the kinase inhibitory activity described for some of the compounds [27,33,34]. The fulfillment of the structural requisites of the pharmacophore model previously developed for β’–σ PPI inhibitors might facilitate the design process.

In addition to their prospective use as novel antibiotics, PPI inhibitors have already entered the drug market in important clinical areas such as cancer therapeutics, and many other candidate PPI drugs are currently being evaluated in clinical trials [30]. Our PPI inhibitor discovery workflow, here applied to bacterial RNAP holoenzyme assembly inhibition, can be easily adapted to other bacterial and eukaryotic PPIs. Indeed, the advantage of using yeast as an in vivo screening test tube is even more relevant in the case of proteins that are toxic to (and actually kill) bacterial cells, such as type II toxin–antitoxin systems [32,35] that are emerging as highly promising targets for the development of new antibacterial agents [5].

In this context, we believe that the streamlined screening workflow validated in the present work may represent an effective and highly versatile tool for the identification of new PPI-interfering hit compounds to be developed into novel therapeutics.

## 4. Materials and Methods

### 4.1. Chemicals

Compound **4** was purchased from ChemDiv (San Diego, CA, USA). The NanoGlo Luciferase Assay Substrate was purchased from Promega (Madison, WI, USA), Polymyxin B nonapeptide (PMBN), dimethyl sulfoxide (DMSO), bovine serum albumin (BSA), Tween-20, phosphate-buffered saline (PBS) were purchased from Sigma-Aldrich (St. Louis, MO, USA). D(+)-glucose, D(+)-galactose, D(+)-raffinose, yeast nitrogen base without amino acids, Bacto peptone, Bacto yeast extract and the different amino acid complements were purchased from Formedium™ (Swaffham, Norfolk, England).

### 4.2. yBRET Assay

The yeast BRET assay was conducted as previously described in Sartini et al. [18]. The CH region of β′ (aa 1–334), the target of the inhibitor, was fused with the acceptor protein Yellow Fluorescent Protein (YFP) and constitutively expressed, while full-length σ^70^ was fused with the donor protein, the deep-water shrimp-engineered luciferase NanoLuc (NLuc). Its expression was under the control of the yeast GAL1 promoter. The yeast strain *S. cerevisiae* erg6Δ (BY4742 background: MATa; his3Δ1; leu2Δ 0; met15Δ 0; ura3Δ 0; YML008c::kanMX4) (Open Biosystems, Huntsville, Alabama, USA) was transformed with the NLuc-σ^70^ p415 and β’ (aa 1–334)-YFP p416 vectors.

For screening, yeast cultures were grown until an OD_600_ of 0.8 and then aliquoted (25 μL per well) into empty 96-well plates (LumiNunc™ F96 MicroWell™; VWR International, Radnor, PA, USA) prefilled with the test compounds dissolved in DMSO (1.5 μL/well) at a final concentration of 20 μM. To induce expression from the GAL1 promoter, 25 μL of SR -Ura -Leu medium containing galactose at a final concentration of 0.5% was added and the plates were incubated in an orbital shaker (130 rpm) at 30 °C for 2 h. 

Luminescence was measured with a microplate reader (TriStar^2^ LB 942; Berthold Technologies, Bad Wildbad, Baden-Württemberg, Germany), using high-efficiency BRET filters (480 nm and 530 nm). The BRET ratio was calculated by dividing the signal measured at 530 nm by the signal measured at 480 nm. The BRET signal was calculated as the BRET ratio subtracted from the background BRET ratio (i.e., the BRET signal measured in the presence of the donor protein alone) and multiplied by 1000 to express the results as milliBRET (mBRET).

### 4.3. Competitive ELISA Assay

The production of recombinant proteins, the standard ELISA, and the “reverse” ELISA were performed following the protocols described in Sartini et al. [18]. The full-length sequence coding for *E. coli* σ^70^ was cloned into a modified pET28-b in-frame with an N-terminal 6xHis tag-sequence and was expressed in LB medium for 3 h at 30 °C following addition of 1 mM isopropyl-β-D-thiogalactopyranoside (IPTG). After sonication the lysate was subjected to metal affinity chromatography purification on HIS-Select^®^ Cobalt affinity Gel (Sigma-Aldrich, St. Louis, MO, USA) column and exchanged into 20 mM NaH2PO4, 150 mM NaCl (pH 8.0) containing 20% (*v/v*) glycerol. 

The RNAP β’ 1–334 aa. coding sequence was cloned into a modified pGEX4-T3 expression vector (Amersham Biosciences, Amersham, Buckinghamshire, England) and was expressed for 24 h at 20 °C under auto-induction conditions (Auto Induction Medium, AIM-LB broth base; ForMedium™, Swaffham, Norfolk, England). Due to the failure of affinity purification on a glutathione-Sepharose column, the unfractionated whole cell supernatant was used directly as a GST-tagged source for ELISA. 

For the standard ELISA, His-tagged- σ^70^ was diluted to 250 nM with PBS and 100 μL of the resulting solution was added to the wells of SpectraPlate-96 HB microtitre plates (PerkinElmer, Waltham, MA, USA). After an overnight incubation at 4 °C, the wells were washed with PBS and blocked at room temperature for 2 h with 400 μL of 1% (*w/v*) BSA dissolved in PBS. Subsequently, 100 μL of supernatant diluted in PBS containing 50 nM GST-β′ (aa 1–334) was incubated for 10 min at 37 °C with 2.5 μL of each compound dissolved in DMSO at different concentrations (or with 2.5 μL of DMSO). The addition first of the polyclonal rabbit anti-GST primary antibody (1:2000 in PBS) (Abcam, Cambridge, Cambridgeshire, England) and after incubation, of the horseradish peroxidase-conjugated goat anti-rabbit secondary antibody diluted 1:20,000 in PBS (Invitrogen™ by Life Technologies™, Carlsbad, CA, USA) allowed the detection of the interaction using the substrate for 2,2’-Azino-bis (3-Ethylbenzthiazoline-6-Sulphonic Acid) peroxidase (ABTS; SeraCare Life Sciences, Milford, MA, USA). The signal resulting from the oxidation of ABTS was determined by measuring the absorbance at 415 nm with a microplate reader (iMark™ Microplate Absorbance Reader; Bio-Rad, Hercules, CA, USA).

For the “reverse” ELISA, after the overnight plate coating with His-tagged-σ^70^ and blocking step, 50 μL of PBS, containing 2.5 μL of each compound dissolved in DMSO (or DMSO as “vehicle control”) were added to the σ^70^ precoated wells and incubated for 20 min at 37 °C. Then 50 μL of β’ supernatant containing 100 nM GST-β′ (1–334) were added and further incubated at RT for 1 h. Primary and secondary antibodies’ incubations and detection conditions were the same as for standard ELISA.

### 4.4. Antibacterial Activity Assays

Growth inhibition tests and minimum inhibitory concentration (MIC) assay on the gram-positive *B. subtilis* WB800N strain (nprE aprE epr bpr mpr::ble nprB::bsr Δvpr wprA::hyg cm::neo; NeoR) (MoBiTec GmbH, Lotzestraße, Göttingen, Germany) and on the gram-negative nonpathogenic *E. coli* DH10 T1R strain (F-mcrA Δ(mrr-hsdRMS-mcrBC) Φ80lacZ ΔM15 ΔlacX74 recA1 endA1 araD139Δ(ara, leu)7697 galU galK λ-rpsL nupG) (Invitrogen, Waltham, MA, USA) were performed as described in Sartini et al. [18]. 

For growth inhibition assays, bacteria were grown to exponential phase (OD_600_ = 0.5–0.6) and then diluted 100-fold with LB. PMBN (2 μg/mL) for *E. coli* was added at this stage. Yeast strain W303-1A (leu2-3,112 trp1-1 can1-100 ura3-1 ade2-1 his3-11,15) [36] was grown at 30 °C in YPD to an OD_600_ of 0.5. The growth of the bacterial (or yeast) cells was monitored for 8 h in the presence of the candidate compounds dissolved in DMSO (final concentration of 5%) or an equivalent amount of DMSO as a negative control (“vehicle”). Growth inhibition assays were conducted in triplicate in 96-well plates (Microtest Plate 96 Well, F, flat base, Sarstedt, Nümbrecht, North Rhine-Westphalia, Germany) by monitoring cell growth (OD600) every 30 min with a microplate reader (TriStar^2^ LB 942; Berthold Technologies, Bad Wildbad, Baden-Württemberg, Germany) for bacteria and every 1 h for yeast.

Antimicrobial activity was determined by means of the minimum inhibitory concentration (MIC) assay, according to the Clinical & Laboratory Standards Institute (CLSI) guidelines described in ([37]). The assays were performed in 96-well microplates in 20% MH broth in PBS. A 2.5 × 105 CFU/mL medium-phase bacterial suspension in 20% MH broth was used and the microplates were incubated at 37 °C for 24 h. The MIC value was considered to be the lowest compound concentration that resulted in complete inhibition of visible bacterial growth after an appropriate incubation time. The results were derived from at least three independent experiments conducted in duplicate.

### 4.5. In Vitro Transcription Assay

A fluorescence-detected RNAP inhibition assay with the profluorescent substrate γ-[2′(2-benzothiazoyl)-6′-hydroxybenzothiazole)-ATP (γ-BBT-ATP, Jena Bioscience, Jena, Thuringia, Germany) was performed to verify the ability of the selected compounds to inhibit the specific transcription activity of bacterial RNA polymerase, using *E. coli* RNAPc (New England BioLabs, Ipswich, MA, USA) and recombinant His-tagged-σ^70^ for holoenzyme reconstitution. 

Individual compounds (specified in the main text) were added to RNAPc (prior to σ^70^ addition and holoenzyme reconstitution) at the required concentration in order to be tested under noncompetitive inhibition assay conditions. To this end, candidate compounds or equivalent amount of DMSO (4% final concentration) were first incubated with 87.5 nM RNAPc and 2 µL of 5× *E. coli* RNA Polymerase Reaction Buffer at RT for 15 min, followed by the addition of 87.5 nM σ^70^ and a further 15 min incubation at RT. Two nM of a circular DNA template (pDSP) [38] were then added to a final volume of 7 µL and the resulting reaction mixture was incubated at 37 °C for 15 min. The pDSP template is a 6135 bp vector harboring two identical λpR promoters upstream of engineered, cytosine-lacking (C-less) DNA regions. When CTP is omitted from the reaction mixture, transcription reinitiation is prevented, and this allows for the single-round transcription of 24 nt and 70 nt long RNAs. Accordingly, transcription was started with the addition of a CTP-lacking mix of unlabeled NTPs (1 µL of 1 mM UTP, 1 µL of 1 mM GTP) plus 1 µL of γ-(BBT)-ATP 250 µM and incubated for 10 min. Profluorescent BBT-diphosphate produced during reactions was hydrolyzed to fluorescent BBT by addition of 0.5 µL of CIP and 1.2 µL of CIP buffer to a final volume of 12 µL and further incubated at 37 °C for 20 min.

Ten µL of the reaction was transferred to a black 384-well plate (PE384fw_ProxiPlate) and fluorescence emission was read with Spark 10M, Tecan (Männedorf, canton of Zürich, Switzerland) (excitation wavelength 422 nm; emission wavelength 590 nm).

### 4.6. Alignment of New Compounds on Indolyl-Urea **4**

The reference compound **4** in the conformation fitting the 5-points pharmacophore model is used as the query for a shape-similarity performed with Phase [39,40] of the Schrödinger suite 2021-1. The input query is converted into a collection of pharmacophoric sites, each represented by a sphere of 2 Å. According to Phase Shape procedure, the conformations of each new inhibitor (B) are generated on-the-fly and their Phase pharmacophoric features are aligned on those of the reference compound (A) through a shape screening similarity [41]. The similarity score was computed as the sum of pairwise atomic overlaps (O_AB_), considering the overlaps of sites sharing the same pharmacophore feature type, normalized to the largest self-overlap (either O_AA_ or O_BB_), according to the formula:

$${\mathrm{Sim}}_{\mathrm{AB}}=\frac{O_{\mathrm{AB}}}{\max\left(O_{\mathrm{AA}},O_{\mathrm{BB}}\right)}$$

The alignment with the highest similarity score was retained and energy-minimized for each new inhibitor. The minimization was executed with OPLS4 force field [42] implemented in MacroModel 13.1 [43] in the GB/SA continuum solvent model (implicit water) [44] by applying the Polak–Ribière conjugate gradient method until a gradient of 0.01 kJ·mol^−1^·Å^−1^ was reached [45].

## Figures and Tables

**Table 1:** Structure, antimicrobial activity, and β′–σ^70^ binding inhibition capacity of reference and hit compounds. Data are the mean of three replicates.

Library	Compound	Growth Inhibition at 100 μM (8 h)			MIC (μM)		Binding Inhibition
		*B. subtilis*	*E. coli* ^b^	*S. cerevisiae*	*B. subtilis*	*E. coli* ^b^	IC_50_ μM ± SD
**Reference**	**4 ^a^**	73%	91%	0%	50	25	8.09 ± 1.1 ^c^
**ReCC**	**5**	19%	92%	1%	>100	1.56	15.11 ± 1.96
	**7**	13%	66%	7%	>100	0.78	8.56 ± 1.82
**ICBMS**	**9**	24%	24%	0%	>100	>100	13.93 ± 1.86
	**13**	40%	29%	0%	50	50	16.35 ± 1.8
**PKRC**	**19**	37%	0%	0%	100	>100	40 ± 4.2
	**20**	15%	30%	0%	100	12.5	80 ± 6.56

^a^ Compound **4** (referred to as **43** in [18]) was used as reference. ^b^ Experiments in *E. coli* were conducted in the presence of PMBN (2 μg/mL). ^c^ Taken from reference [18].

## Data Availability

Data is contained within the article or Supplementary Material.

## Supplementary Materials

The following supporting information can be downloaded at: https://www.mdpi.com/article/10.3390/antibiotics11101449/s1, Figure S1: RNA polymerase β’–σ^70^ PPI interaction inhibitors; Figure S2: Visual scheme representing the drug-discovery workflow; Table S1: β’–σ^70^ binding inhibition of yBRET-selected compounds measured through ELISA assay performed at a fixed concentration of 100 μM.
