# Supplementary material for: An Optimized Workflow for the Discovery of New Antimicrobial Compounds Targeting Bacterial RNA Polymerase Complex Formation
Source: Antibiotics (Basel). 2022 Oct 21;11(10):1449. doi: 10.3390/antibiotics11101449 (PMC9598883; doi:10.3390/antibiotics11101449)
Supplement: Supplementary file 1 [file antibiotics-11-01449-s001.zip › antibiotics-1971715-supplementary.pdf]

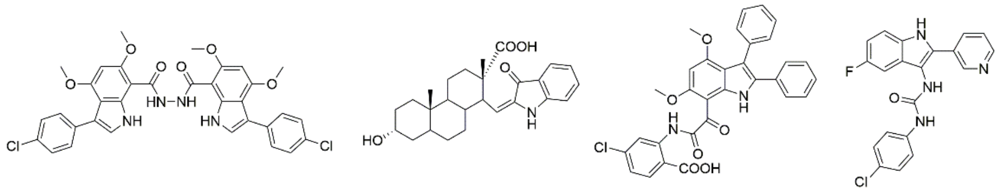

GKL003, **1** C5, **2** **3** **4**

Figure S1. RNA polymerase β′-σ[70] PPI interaction inhibitors.

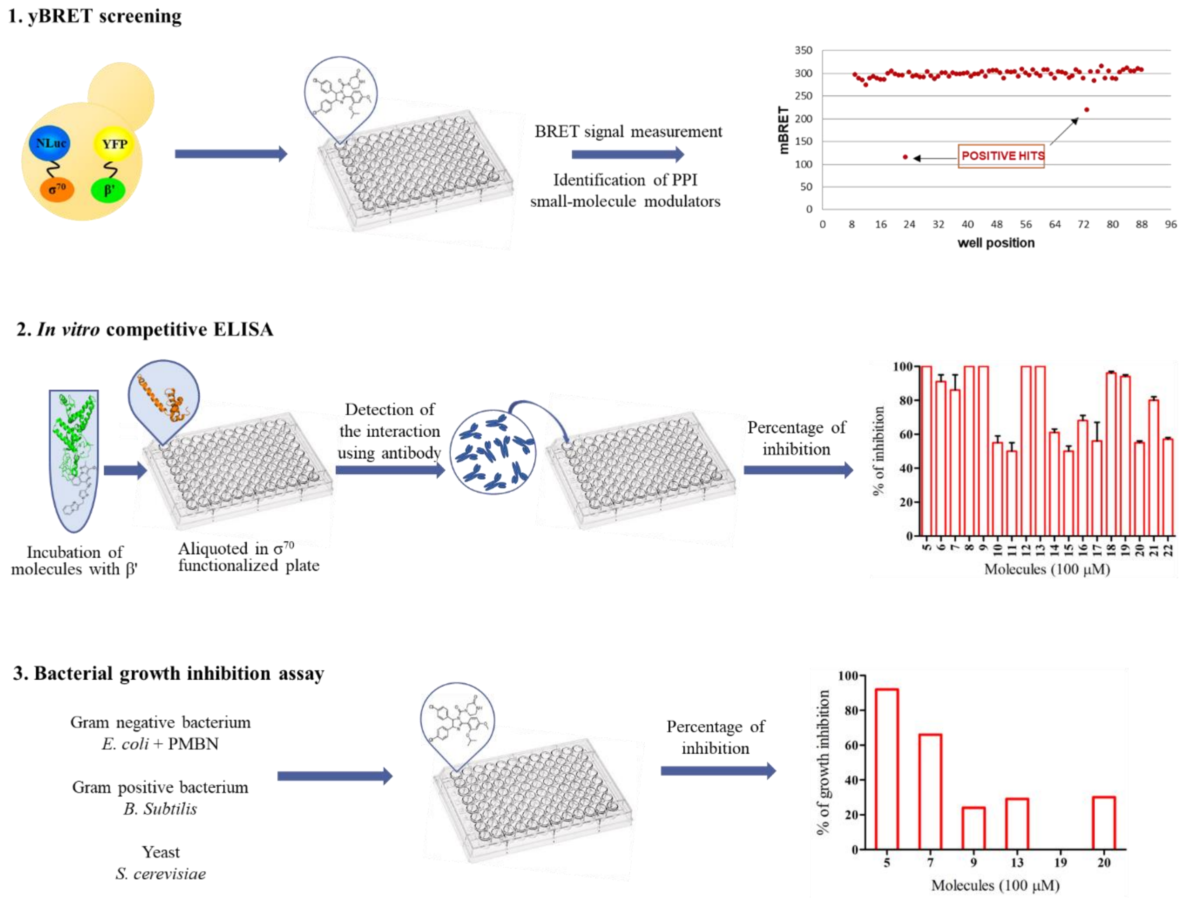

Figure S2. Visual scheme representing the drug-discovery workflow.

Table S1. β′-σ^{70} binding inhibition of yBRET-selected compounds measured through ELISA assay performed at a fixed concentration of 100 μM. Data are the mean of three replicates; SD is reported.

Library	Molecule	Structure	ELISA 100μM
**ReCC**	**5**	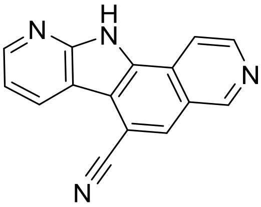	100
	**6**	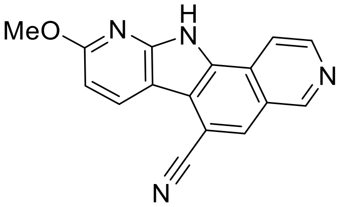	91±4
	**7**	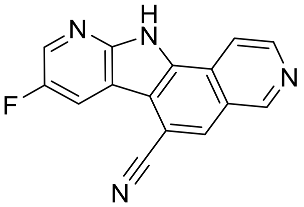	86±9
	**8**	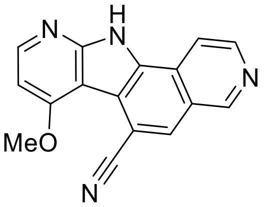	100
**ICMBS**	**9**	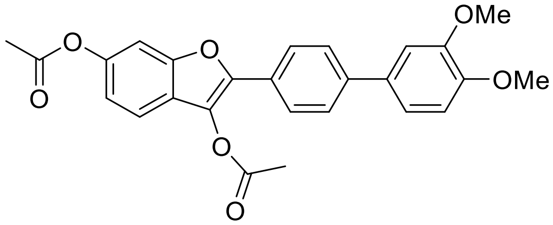	100
	**10**	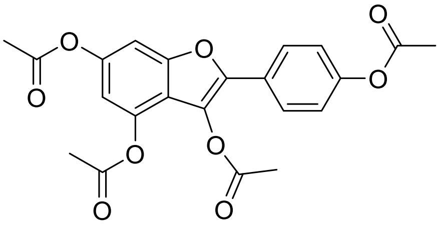	55±4
	**11**	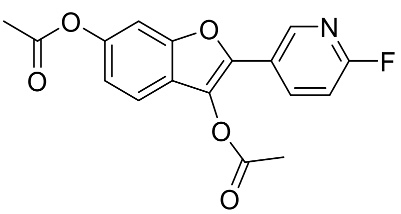	50±5

Library	Molecule	Structure	ELISA 100μM
	**12**	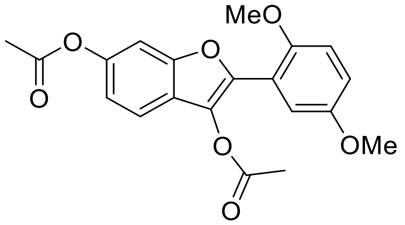	100
	**13**	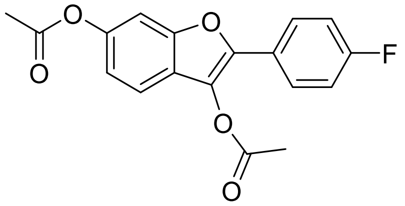	100
	**14**	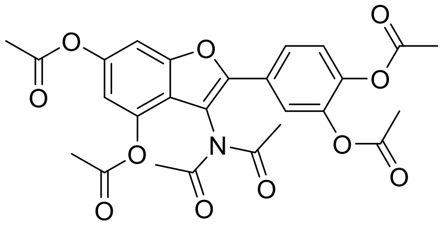	61±2
**PKRC**	**15**	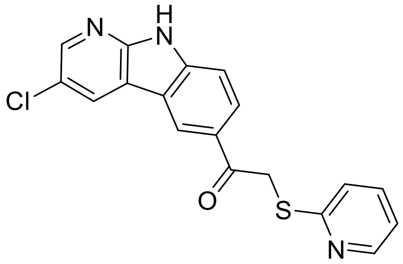	50±3
	**16**	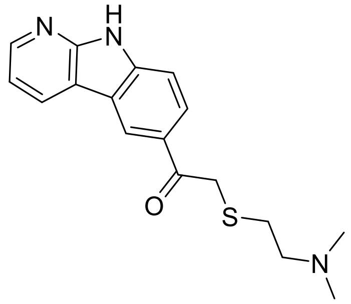	68±3
	**17**	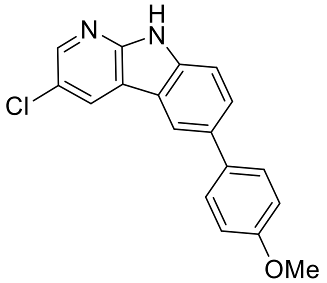	56±11
	**18**	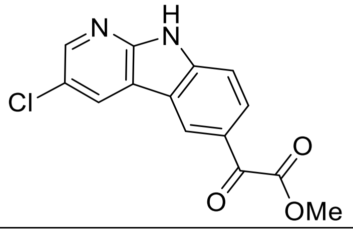	96±1

Library	Molecule	Structure	ELISA 100μM
	**19**	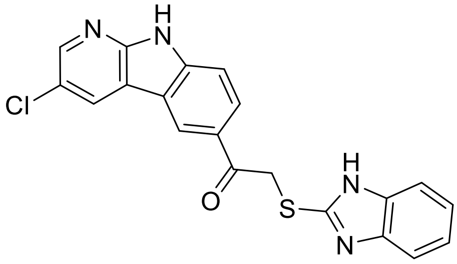	94±1
	**20**	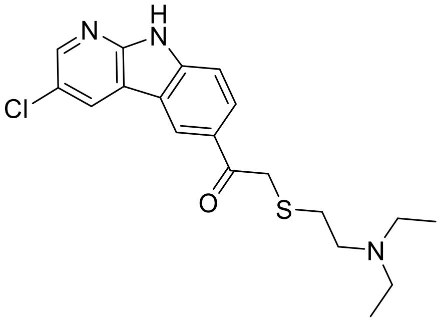	55±1
	**21**	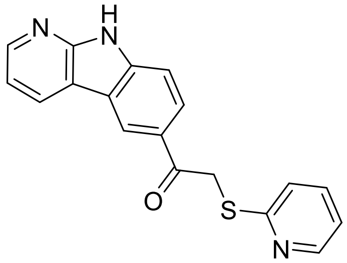	80±2
	**22**	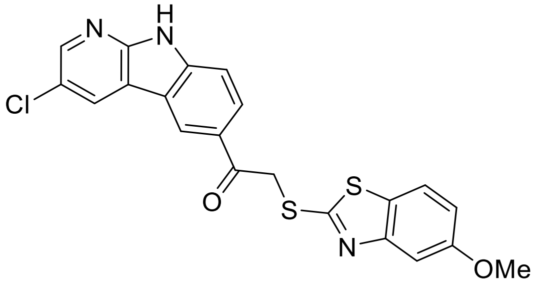	57±1
